# Implementation and evaluation of a rural community-based pediatric hearing screening program integrating in-person and tele-diagnostic auditory brainstem response (ABR)

**DOI:** 10.1186/s12913-018-3827-x

**Published:** 2019-01-03

**Authors:** Vidya Ramkumar, Roopa Nagarajan, Vanaja C. Shankarnarayan, Selvakumar Kumaravelu, James W. Hall

**Affiliations:** 1 Department of Speech, Language and Hearing Sciences, Sri Ramachandra Institute of Higher Education and Research, Porur, Chennai, Tamil Na du-400116 India; 2 Department of Audiology & Speech Language Pathology, Bharati Vidyapeeth Deemed University, Pune, India; 3 Department of Neurosurgery, Chairman Telemedicine, Sri Ramachandra Institute of Higher Education and Research, Chennai, India; 4 Department of Audiology, Nova Southeastern University, Fort Lauderdale, USA; 5 Department of Communication Pathology, University of Pretoria, Pretoria, South Africa

**Keywords:** Community-based program, Paediatric hearing screening, Tele-audiology, Tele-auditory brainstem response

## Abstract

**Background:**

In an attempt to reach remote rural areas, this study explores a community-based, pediatric hearing screening program in villages, integrating two models of diagnostic ABR testing; one using a tele-medicine approach and the other a traditional in-person testing at a tertiary care hospital.

**Methods:**

Village health workers (VHWs) underwent a five day training program on conducting Distortion Product Oto Acoustic Emissions (DPOAE) screening and assisting in tele-ABR. VHWs conducted DPOAE screening in 91 villages and hamlets in two administrative units (blocks) of a district in South India. A two-step DPOAE screening was carried out by VHWs in the homes of infants and children under five years of age in the selected villages. Those with ‘refer’ results in 2nd screening were recommended for a follow-up diagnostic ABR testing in person (Group A) at the tertiary care hospital or via tele-medicine (Group B). The overall outcome of the community-based hearing screening program was analyzed with respect to coverage, refer rate, follow-up rate for 2nd screenings and diagnostic testing. A comparison of the outcomes of tele-versus in-person diagnostic ABR follow-up was carried out.

**Results:**

Six VHWs who fulfilled the post training evaluation criteria were recruited for the screening program. VHWs screened 1335 children in Group A and 1480 children in Group B. The refer rate for 2nd screening was very low (0.8%); the follow-up rate for 2nd screening was between 80 and 97% across the different age groups. Integration of tele-ABR resulted in 11% improvement in follow-up compared to in-person ABR at a tertiary care hospital.

**Conclusions:**

Non-availability of audiologists and limited infrastructure in rural areas has prevented the establishment of large scale hearing screening programs. In existing programs, considerable challenges with respect to follow-up for diagnostic testing was reported, due to patients being submitted to traveling long distance to access services and potential wage losses during that time. In this program model, integration of a tele-ABR diagnostic follow-up improved follow-up in comparison to in-person follow-up. VHWs were successfully trained to conduct accurate screenings in rural communities. The very low refer rate, and improved follow-up rate reflect the success of this community-based hearing screening program.

**Electronic supplementary material:**

The online version of this article (10.1186/s12913-018-3827-x) contains supplementary material, which is available to authorized users.

## Background

Community-based approaches are being explored in various disciplines to provide health and rehabilitation services to narrow disparities [1] between urban and rural populations, and semi-urban areas with limited resources. The World Health Organization recommends integrating ear and hearing care into community-based rehabilitation programs as it can improve coverage, especially in rural areas [2], where births are often in homes or primary health clinics [3].

Community-based programs have the advantage of increased sustainability, as programs can be designed utilizing existing resources that are accessible to all members of a community. The involvement of local community leaders and volunteers reinforces community-based programs [4]. In hearing health provision, trained community-health workers can generate awareness in the community, mobilize families for screenings and follow-ups, and guide families through the rehabilitation process [5].

Newborn hearing screening (NHS) programs were implemented in India as research initiatives since 1970’s [6–8]. Since then, a handful of hospital-based programs were also established and have been on the increase year after year [9–13]. However, the reach of these programs has been restricted to a very small section of society. In 2006, the Ministry of Health and Family Welfare, Government of India, launched the ‘National Programme for Prevention and Control of Deafness’ (NPPCD) as a step to promote early identification of congenital and acquired hearing loss. Under this programme, both institution-based screenings and community-based screenings were implemented. At the grassroots level, health workers, anganwadi^1^ workers, and birth attendants were trained to generate awareness regarding hearing loss prevention and to facilitate early detection using behavioural measures at immunization clinics and through home visits. To target older children with hearing loss, school screening camps were to be conducted by doctors with the assistance of primary school teachers. Diagnostic evaluations and management of children referred from the community and school screenings were carried out by an ENT specialist, audiologist or audiometrician at a district hospital [14]. This program integrated primary ear care with primary and district health systems, thus having the potential to reach both urban and rural populations. The program was piloted in 25 districts in 2006 and was expanded to 192 districts by 2013 [15]. However, impact assessments suggests that lack of infrastructural facilities, as well as shortages of audiologists and equipment in district hospitals plagued the program in several states [16, 17]. Such shortcomings in human resources and infrastructure at rural centres makes newborn hearing screening unviable, as parents are unlikely to travel to distant centres for diagnostic testing, due to transportation costs, lost wages or for cultural reasons. Such shortcomings maybe overcome by providing diagnostic testing services remotely using tele-practice.

Tele-practice, which is the provision of health services from one location to another using telecommunication as a medium, offers real benefits in a country as vast as India where the majority of the population lives in remote areas. One clear advantage of tele-practice for service provision is the significant reduction in cost, as it averts patients’ expenses towards travel, accommodation, and treatment in city hospitals [18]. Additionally, from an administrative perspective, the cost of infrastructure development, personnel and equipment can be significantly minimized.

In an attempt to reach rural areas, this study explores the combination of a community-based pediatric hearing screening program in remote rural villages integrating two models of diagnostic auditory brainstem response (ABR)^2^ testing; i) using tele-medicine approach ii) in-person at a tertiary care hospital. The study aimed to evaluate the efficacy of a community-based pediatric hearing screening program with integrated tele- and in-person diagnostic follow-up.

The audiological equipment used for this study was sponsored by GSI Inc. USA, the mobile telemedicine van with satellite connectivity was provided by the Indian Space Research Organization (ISRO) and all the recurring expenses including salary for health workers and technicians was funded by the Indian Council of Medical Research.

## Method

This study was approved by the Institutional Ethics Committee of Sri Ramachandra University.

### Study design

**Fig. 1:**
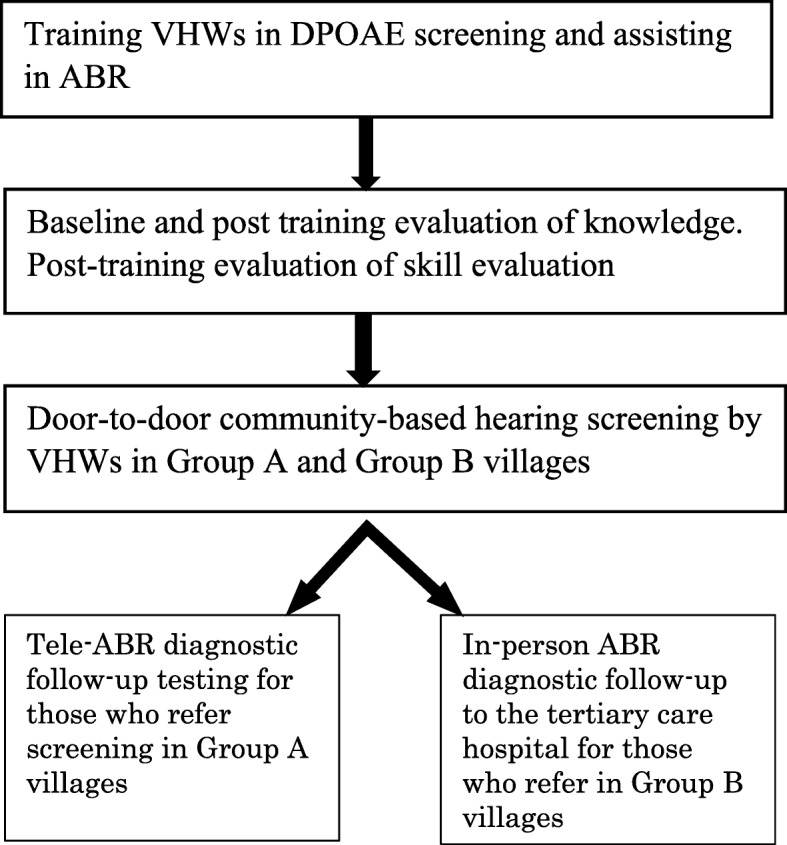
Schematic representation of the steps involved in the study

Prospective cohort. A schematic representation of the steps involved in the study is shown in Fig. 1.

#### Training village health workers to screen hearing using distortion product Oto acoustic emissions^3^ (DPOAE) and assist in ABR testing

Seven Village Health Workers (VHWs) were recruited for training through the non-profit, Rural Women’s Social Education Centre (RUWSEC) located in the community. All VHWs were women with minimum five years of field work experience, having minimum eighth grade education, and demonstrated good communication skills.

##### Training

Training was conducted in the local language (Tamil) and included PowerPoint presentations, hand-outs and videos. The World Health Organization: Primary Ear and Hearing Care Training Resource and National Centre for Hearing Assessment and Management’s educational and training resources were used as the framework for the development of these materials.

VHWs underwent five training sessions of 6 h each, over the course of two weeks. Information was provided on the relevance and need for screening, as well as of screening methods. Concepts of false positives and false negatives were explained. VHWs were instructed to ensure that the environment was conducive for DPOAE screening and were encouraged to screen while the infant was asleep. For DPOAE screening, training was conducted through video material and live demonstrations with hands-on training on five adults. Training in tele-ABR assistance for preparing skin, placing electrodes and transducer also included video presentations, live demonstrations, and hands-on training on baby mannequins and on five adults.

##### Evaluation

**Fig. 2:**
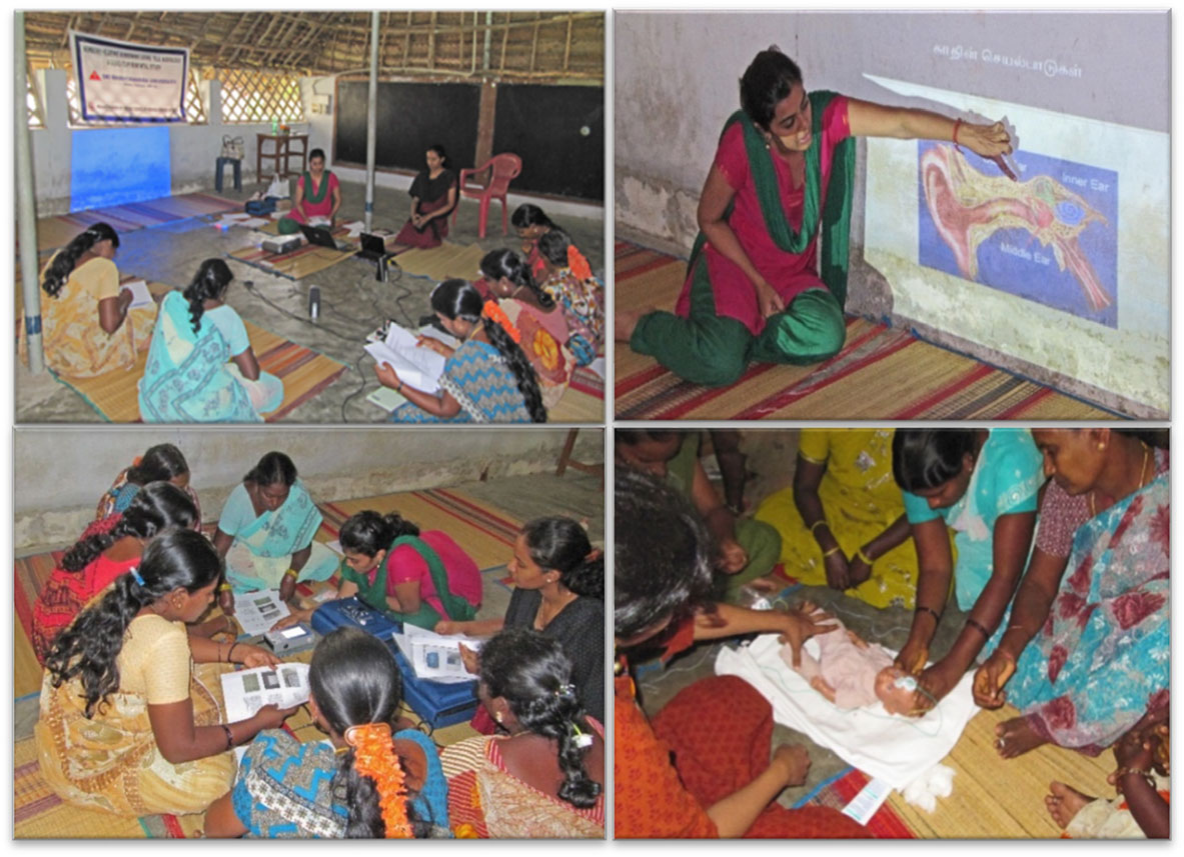
Training program (top left) baseline evaluation (top right) power point presentation by audiologist regarding anatomy of ear (bottom left) training on DPOAE screening (bottom right) training VHWs in ABR assistance

Performance in the post training evaluation determined recruitment of VHWs in the screening program*.* Follow-up evaluations (6 months and 1.5 years post-training) were also conducted to assess retention of knowledge and skill. Knowledge was evaluated at baseline and post-training using 15 multiple-choice questions (Additional file 1). The questions pertained to age of screening, risk factors of hearing loss, methods of hearing screening, interpretation of screening results and consequences of hearing loss. Skill was evaluated post-training using an objective structured clinical examination (OSCE) format, in which, VHWs performed the screening process and assisted in ABR on one adult each. Agreement in DPOAE screening result between audiologist and VHW was assessed on 10 infants and 20 adult ears. Images from the training program are shown in Fig. 2.

#### Implementation of community-based hearing screening program

##### Location of screening program

**Fig. 3:**
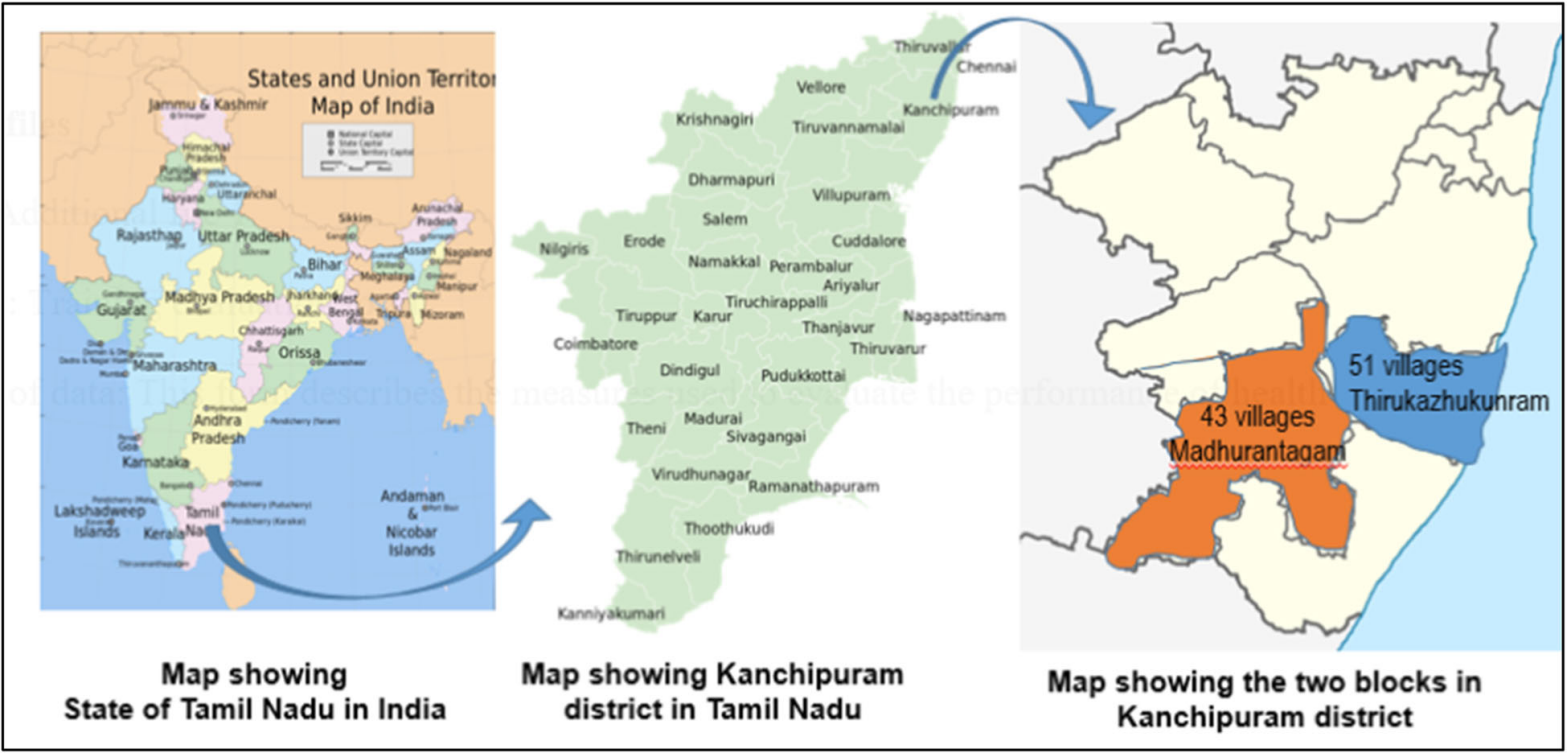
Map depicting location of the program. Map of sates and union territories of India [45], map of Tamil Nadu [46], map of Kanchipuram district [47]

Fifty-one villages and hamlets with an estimated population of 32,560 in Thirukazhukunram block (Group A) and 43 villages and hamlets with an estimated population of 33,642 in Madhurantagam block (Group B) were selected for the program. The location of the screening program is shown in Fig. 3. Villages were approximately equal in distance from the tertiary care hospital. The population of Group A and Group B were nearly equal. VHWs proximity to prospective villages was a factor in village selection.

##### Sample

All infants^4^ and young children^5^ up to five years of age residing in the selected villages were included. Both written and verbal consent from parent was obtained prior to hearing screening.

##### DPOAE screening settings

Two automated GSI Audioscreener+ hand-held devices were used for screening. DPOAE screening was conducted in homes of the infants and young children. The DPOAE protocol was based on results of a pilot study in the same community. The maximum ambient noise levels did not exceed 50dBA. DPOAE screening was conducted at 2, 3, and 4 kHz with stimulus intensity level of 55 dB SPL (L2) and 65 dB SPL (L1), and the environment was set as “noisy”. The automated algorithm for ‘pass’ criteria was 6 dB SNR at two out of three frequencies.

##### Hearing screening

Six VHWs were recruited for the screening program. Four VHWs conducted DPOAE screening in the community, two VHWs each in Group A and Group B villages. Two other VHWs supervised the program and mobilized community participation. VHWs informed parents about the hearing screening program through balwadi^6^ teachers as well as through personal visits. VHWs documented demographic details and high risk factors while conducting the first screening. If a child “referred” in the first screening, re-screening was conducted after two weeks.

##### Diagnostic confirmation of hearing loss using ABR

Children with “refers” on 2nd screenings were directed to an audiologist for diagnostic testing. Diagnostic testing was completed using one of two testing models: (i) auditory brainstem response (ABR) testing by the audiologist at the/a tertiary care hospital, or (ii) tele-ABR testing by the audiologist from the tertiary care hospital at the rural location via remote computing.

GSI Audera was used for acquiring ABR waveforms. ABR was recorded using click stimuli (0.1 ms) with monaural stimulation at a rate of 33.1/s in rarefaction polarity. Rate of stimulation was reduced to 11.1/s in newborn and infants if ABR waves had poor morphology. Standard recording parameters were used. Intensity was varied to identify the lowest level at which replicable waveforms could be obtained.

ABRs were analyzed for wave morphology, repeatability and peak latency. Presence of peak V up to 30 dB nHL was considered normal hearing. For tele-ABR, presence of peak V up to 40 dB nHL was accepted as normal when ambient noise levels increased in the mobile tele-van due to the use of a power generator.

##### In-person diagnostic testing

ABR was conducted by an Audiologist (first author) in the Audiology clinic at the tertiary care centre.

##### Tele-diagnostic testing

**Fig. 4:**
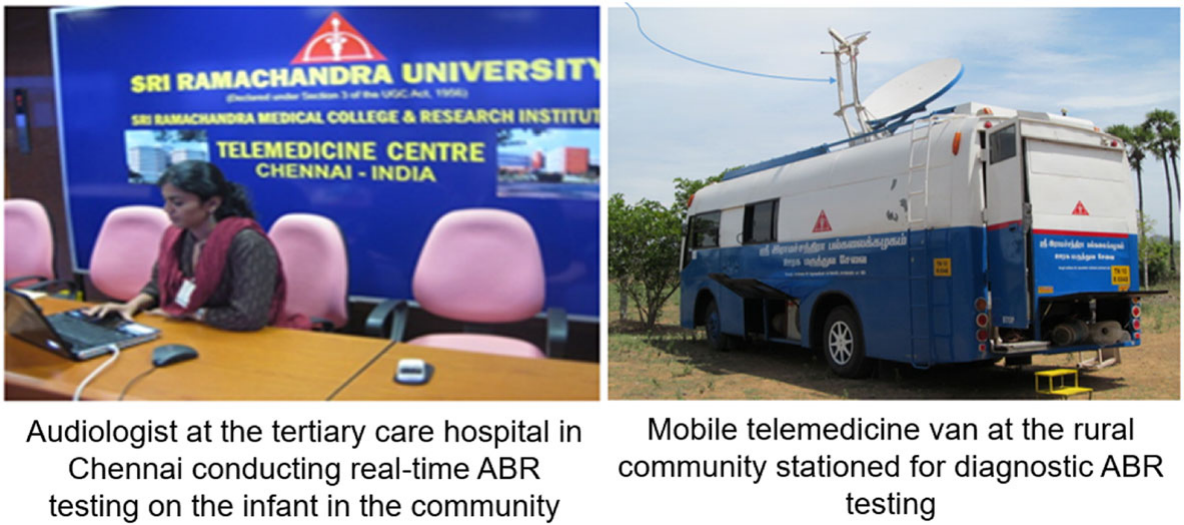
Schematic representation of tele-audiological testing using mobile tele-van

Tele-diagnostic ABR testing was conducted using a mobile telemedicine van with satellite connectivity in rural areas that lacked internet penetration or otherwise with broadband internet. This testing was conducted in real time by an audiologist at the tertiary care hospital by remotely accessed equipment. The trained VHWs prepared the child for testing (electrode placement, positioning child and ensuring that the child was asleep throughout testing). A tele-technician set up the equipment and established satellite / broadband internet connectivity. Detailed description of the mobile telemedicine van with satellite connectivity and validation of tele-ABR protocol is published in Ramkumar et al. [19]. A schematic representation of tele-ABR diagnostic testing is shown in Fig. 4.

### Analysis

The outcome of training conducted for VHWs was evaluated using the Friedman test of repeated measures and percentage analysis. The overall outcome of the community-based hearing screening program was analyzed with respect to coverage rate, refer rate, follow-up rate for 2nd screening and diagnostic testing, alongside a comparison of outcomes of tele-versus in-person diagnostic ABR follow-up.

## Results

### Outcomes of training

#### Evaluation of knowledge

**Fig. 5:**
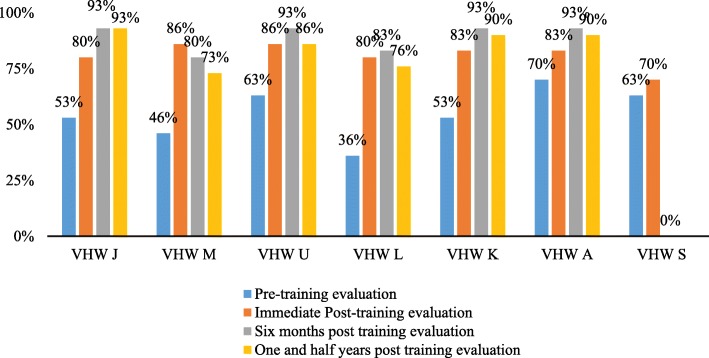
Performance of VHW pre and post training evaluation in knowledge on ear and hearing care

A benchmark criteria of 80% scores in knowledge assessment was set for recruiting VHWs for the screening program. On pre-training evaluations, VHWs scored between 35 and 70%. Figure 5 reflects that all VHWs achieved between 80 and 86% scores in the immediate post evaluation.

VHWs were encouraged to review the manual and videos to refresh their knowledge and maintain quality of service. Six months post-training, a secondary evaluation was conducted where all VHWs obtained a minimum of 80% scores. In the evaluation conducted one and a half years post training, two VHWs obtained less than 80%. In such cases, information provided during the training were recapitulated once again.

On the Freidman test of repeated measure, a significant difference (at α = 0.01) was obtained between baseline scores and immediate post, six months post and one and half years post training (F = 19.507, df (3,15), *P* = 0.00002). Prior to training, VHWs were aware of some of the risk factors of hearing loss such as trauma to the ear, perforated ear due to use of hair pins or other sharp objects, excessive noise exposure and consangnous marriage was reported as a risk factor for all disabilities. The post-training evaluations showed considerable improvement in the scores obtained by VHWs on questions related to importance of age of identification of hearing loss and effective methods for screening children for hearing loss. VHWs answered questions related to DPOAE screening such as adequate conditions for testing, what information could be obtained through DPOAE screenings, and how to make inferences based off of screening results and messages on DPOAE screeners related to noise and probe fit.

#### Evaluation of skill

A benchmark criteria of 80% scores in skill assessment was set for recruiting VHWs for data collection. All VHWs except VHW S scored above 80% in conducting DPOAE screening and ABR assistance as per the minimum requirement (Table 1).

**Table 1:** Performance of VHWs in spotter identification and in skill evaluation

VHW	Skill evaluation DPOAE screening	Skill evaluation ABR assistance
J	88%	85%
U	88%	100%
M	100%	85%
S	77%	28%
A	100%	85%
K	100%	100%
L	100%	85%

**Fig. 6:**
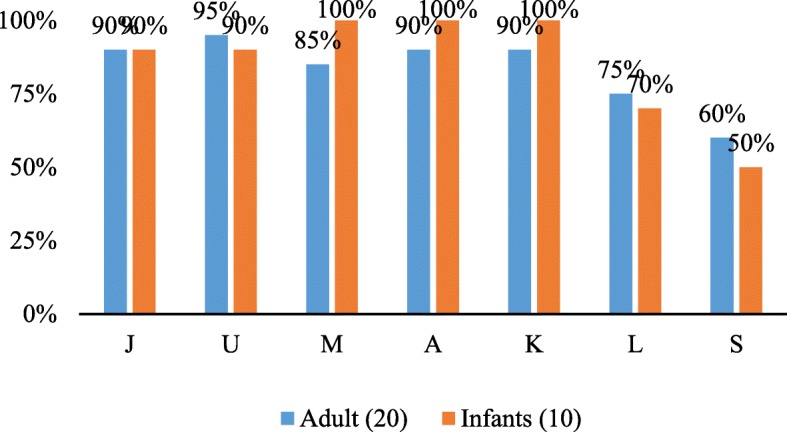
Agreement between VHW and audiologist in results of DPOAE screening

Agreement of results in DPOAE screening between audiologist and VHW was assessed on 10 infant and 20 adult ears. Four VHWs had 90% agreement on both adults and infants. Two VHWs achieved less than 80% agreement (Fig. 6).

All VHWs except VHW S and VHW L obtained above 80% in knowledge, skill and inter-tester reliability. VHW L obtained less than 80% in reliability alone, hence was provided additional training and performance was monitored for two months, before accepting her in the program. VHW S was excluded from the program due to unsatisfactory skill levels despite retraining.

### Community-based hearing screening by VHW for infants and young children

VHWs screened 1335 children (687 infants and 648 young children) from 51 villages in Group A and 1480 children (826 infants and 653 young children) from 43 villages in Group B. Table 3 describes the age distribution of children who underwent screening.

Coverage rate was calculated for the 0–3 months group based on the national birth rate of 20 per 1000 population and the approximate population (30,000) in each group of villages. The average coverage rate was 77% (Group A: 65%; Group B: 90%). Only five mothers did not consent for screening hearing of their child. Time taken for screening ranged from 10 min to 60 min.

VHWs recorded risk factors either from the birth record, if available, or from maternal report. VHWs experienced limitations in obtaining detailed information on level of hyperbilirubinemia, extent of pre-term, and, at times, the exact birth weight from the mothers. Table 2 shows the risk factors of hearing loss present among the infants and young children screened in the community. More than 60% had the risk factor of consangnous marriage. The next common risk factor noted was low birth weight. Hearing loss among those with high risk factors was 8 out of 836 (0.95%).

**Table 2:** High risk factors for hearing loss among infants and young children screened

Significant history	Number of children
Consangnous parents	522
Low Birth Weight	134
Hyperbilirubinemia	43
Family history of Hearing loss	32
NICU admission	29
Total	836

#### Follow-up rate for 2nd screening

The median follow-up rate for 2nd screening was 85%. Mothers remained in their maternal homes often between three to six months after delivery but relocated to their husband’s home after this period. Hence, follow-up rate was poorer for infants between zero to three months despite VHWs conducting door-to-door screening. In the four to five year age group, there were children of nomadic tribes who moved from one location to another and could not be followed up.

#### Refer rate

The median refer rate for the 1st screening was 4.4% and for 2nd screening was 0.8% (Table 3). The 2nd screening refer rate was below 1% for children under three years of age, and less than 2% for children between three and four years of age. A higher refer rate (6.3%) was noted only among four to five-year-old children. It can be noted that the refer rate increased with age.

**Table 3:** ‘Refer rate’ and follow-up rate for second screening in the community-based hearing screening conducted by VHW in 94 villages (Group A and Group B villages)

Age	Children screened 1st screen	Children referred in 1stscreen	1^st^ screen refer rate	Follow up rate for 2nd screening	Children screened in 2nd screen	Children referred in 2ndscreen	2^nd^ screening refer rate
0-3 m	928	19	2.0%	68.4%	13	1	0.1%
4-6 m	226	10	4.4%	80%	8	1	0.4%
7 m-1y	360	14	3.9%	92.8%	13	3	0.8%
> 1–2 y	478	19	4.0%	94.7%	18	3	0.6%
> 2–3 y	403	30	7.4%	100%	30	4	1.0%
> 3–4 y	308	25	8.1%	100%	25	5	1.6%
> 4 -5y	112	12	10.7%	58.3%	7	7	6.3%

#### Follow-up for in-person versus tele-ABR diagnostic testing

In-person ABR was recommended for four children referred in the 2nd screening and three followed up, resulting in 75% follow-up rate. Tele-ABR was recommended for 20 children referred in the 2nd screening and 17 followed-up. In addition, two children who passed the screening but subsequently developed ear infections were also asked to follow up for tele-ABR and both followed up, resulting in 86% follow-up rate. Table 4 shows the number of children referred for 2nd screening and those who followed up for diagnostic testing in each model. The number of children who required in-person follow-up were very few, though tele-ABR follow-up was better than in-person follow-up. This result can be used to construct a hearing screening model for larger cohorts in other geographical areas. The median time between 2nd screening and tele-ABR follow-up was 30 days (10–189 days); and in-person ABR follow-up was 31 (30–36 days).

**Table 4:** Comparison of tele and in-person ABR diagnostic follow up

Age	1st screen	Refer in 1st screen	2nd screen	Missed 2nd screen	Refer in 2nd screen	No. of Tele-ABR	No. of Hearing loss
Group A villages (Tele- diagnostic follow up)							
0–3 m	390	13	9	4	1	1	1
4–6 m	130	7	5	2	1	1	0
7 m-1 yr	167	11	10	1	3	3	1
> 1–2 yr	231	12	11	1	3	3	0
> 2–3 yr	211	19	19	0	3	2	1
> 3–4 yr	159	15	15	0	3	2	1
> 4 -5 yr	47	10	8	2	6	5	1
Total	1335	87	77	10	20	17	5
Group B villages (In-person diagnostic follow up**)**							
0–3 m	538	6	4	2	0	0	0
4–6 m	96	3	3	0	0	0	0
7 m-1 yr	193	3	3	0	0	0	0
> 1–2 yr	247	7	7	0	0	0	0
> 2–3 yr	192	11	11	0	1	0	0
> 3–4 yr	149	10	10	0	2	2	2
> 4 -5 yr	65	2	2	0	1	1	1
Total	1480	42	40	2	4	3	3

Five of seventeen (30%) who underwent tele-ABR were identified with hearing loss, and all three children who underwent in-person ABR (100%) had hearing loss. As a result of this program, two infants and six young children with hearing loss were identified. All children with hearing loss exhibited one or more risk factors. Of the eight children with hearing loss, three were born to parents with second degree consanguineous marriage, four had a family history of hearing loss and three had more than one risk factor including low birth weight, jaundice, preterm birth and/or a family history of hearing loss.

Of the eight infants and young children identified with hearing loss, one child was under three months of age, and was subsequently followed up to rule out neuromaturational delay. Two children with bilateral mild hearing loss were identified at one and two years of age respectively, and were counseled regarding communication strategies and periodic follow-up; one three-year-old child with unilateral mild conductive hearing loss was referred for otolaryngological evaluation at the nearest government hospital. Two children between three and four years of age with bilateral severe to profound hearing loss, and two children between four and five years of age with asymmetrical sensorineural hearing loss were recommended a hearing aid trial. Only two parents followed-up for a hearing aid trial. Both children were fit with appropriate amplification and referred to the nearest rehabilitation centre.

## Discussion

### Training VHWs to screen hearing using DPOAE and assist in tele-diagnostic ABR testing

The personnel who conduct hearing screenings are vital for successful implementation of these programs. Hearing screening programs have routinely trained nurses to conduct OAE and/or ABR screening in hospital-based programs in Western countries. In India, it is often the audiologist who conducts the screening [9, 11, 12, 20]. Under the NPPCD program, grassroots level workers are trained to provide hearing screenings using a high risk checklist and behavioural observation [14]. The limitations of screening hearings using checklists and subjective measures are well documented [17, 21], whereas objective screenings using OAE/AABR are known to have higher sensitivity and specificity [22–24].

In this program, VHWs conducted objective hearing screenings after receiving systematic training and repeated evaluations. Some programs have trained grassroots workers [25, 26] but little is described about the content or manner of training [27, 28]. In this program, VHWs underwent a five-day training program where material was taught on ear anatomy, hearing phsysiology, early identification and hearing loss interventions. Training included demonstrations and hands-on training in DPOAE screening and ABR assistance. Knowledge and skill retention was assessed periodically post-training. Regular training and supervision is recommended to improve health workers’ ability to successfully screen [29], as such regular review of manual, videos and troubleshooting procedures were encouraged. Monthly meetings were used as additional opportunities to review screening protocols, information to be disseminated in the community, documentation, and equipment maintenance. Such refresher training was useful in retention of information and skill, and reflected in the performance of VHWs in the periodic evaluations conducted.

### Outcomes of community-based hearing screening

Hospital-based hearing screening programs for infants are evaluated using the benchmarks given by the Joint Committee on Infant Hearing (JCIH). In developing countries, due to lack of infrastructure and manpower, progress has been made towards community-based hearing screening programs for early identification and intervention as an alternative to ignoring the considerable need for hearing health service delivery across India. Since the perspectives and processes of a community-based approach are unique to each community, it is preferable to formulate guidelines specific to such programs. However, in the absence of appropriate guidelines, JCIH, 2007 was used as a reference to discuss the findings of this program [30].

Hansen et al. (2008) suggests that community health worker-based programs increase the coverage and equity of health service delivery. In this community-based program, the coverage rate (77%) was found to be less than the recommended coverage for hospital-based programs (95%). Information on new births must be accurate for better coverage, and this is possible with information from the Government Primary Health Centers. However, such a collaboration could not be achieved. Since new birth information was obtained from the community’s pre-school teachers, it is possible that some infants were missed. VHWs also had challenges in accessing some localities in the community due to geographical barriers resulting in poor commuting options.

Hospital-based programs have the opportunity to screen a child’s hearing before the child is discharged, which is not relevant to door-to-door screening in the community. However, coverage achieved in this program demonstrates the success in screening infants and young children who would otherwise not have received screening services.

In another community-based hearing screening model, the coverage rate achieved by nurses in a community clinic-based screening in South Africa was 32.4%. Multiple responsibilities shouldered by nurses along with hearing screening was reported to be one of the major reasons for poor coverage [25, 31]. As a result, the researchers recommended appointing dedicated screening personnel as opposed to sharing existing manpower [25]. In this program, dedicated personnel were recruited to conduct screening; this could explain the higher rate of coverage.

The average time required for screening was eighteen minutes. Testing time included settling time as well as time required to complete documentation. Since screening was conducted in the homes of patients, the environment had to be prepared in addition to readying the child for screening. Therefore, it is reasonable to expect the time taken for screening to be more than that of a hospital-based screening. Notably, time taken for screening by VHWs in this program is similar to that reported in studies conducted by health home visits in communities in the UK, where 20 min [32] and 12.2 min were reported [26].

#### Follow-up rate for 2nd screening

Hearing screening programs have ensured higher participation in initial screenings but a major challenge remains in ensuring subsequent follow-up [33–36]. The follow-up rate for 2nd screening in this program is better than those reported in hospital-based hearing screening programs in India [12]. Even in community-based hearing screening programs, the loss to follow-up for 2nd screening was reported to be 52% despite free transportation and no fees [37]. One community clinic-based program in South Africa reports a follow-up rate of 85%, ranging between 50 and 100% across eight community clinics [31]. The results of this study are similar. Better follow-up for 2nd screening in this community-based program can be attributed to the door-to-door screening protocol, where the onus was on the VHW to complete 2nd screenings. It can be surmised from the above studies that when the onus of follow-up is on the parents, follow-up is poorer.

#### Refer rate

It is noteworthy that, in this community-based screening program, the 2nd screening refer rates were very low, except in four to five-year-old age group. The refer rate was lower than the reported refer rate (3 to 19.4%) in other community-based programs from the African region [31, 37]. In these programs, two-step screenings using TEOAE/AABR and DPOAE/DPOAE were conducted in immunization clinics with higher noise levels.

In this program, refer rates increased with age. High refer rates of 6.3% were noted only among four to five-year-old children. Acquired permanent conductive hearing loss was ruled out as children identified with hearing loss in this age group had asymmetrical sensorineural hearing loss. Therefore, the higher refer rate can be attributed to older children’s resistance to being tested and transient middle ear conditions that are more common in this age group. A similar trend was reported with TEOAE screening conducted by auxiliary nurses in a community-based program in Nigeria [37].

VHWs were trained to recognize the “noisy” message that appears in the screener when the environment is not conducive to adequate screening and knew to pause screening. In addition, having dedicated personnel for screenings provided sufficient time to make multiple attempts during 2nd screenings to ensure that the “refer” was not due to ambient noise.

As per JCIH 2007, the “refer” percentage of all infants who fail an initial screening and fail any subsequent rescreening before a comprehensive audiological evaluation should be less than 4%.This suggests that the community-based screening program was successful in meeting the standards set by JCIH (2007) for hospital-based programs. The validity of the screening conducted by VHWs was previously evaluated and the negative and positive predictive values were 98.8 and 27.2% respectively [38]. These findings supplement the success of the program.

#### Follow-up for in-person versus tele-ABR diagnostic testing

The advantage, if any, of a tele- ABR diagnostic testing was studied by comparing it to the traditional in-person ABR follow-up rates. As per JCIH standards for hospital-based programs, 90% of infants requiring diagnostic evaluation should be assessed. The rate of follow-up for tele-ABR nearly achieved this benchmark.

All over the world, achieving 100% follow-up for diagnostics is a challenge. Some hospital-based programs in the US, France, and Malaysia showed higher follow-up rates, between 81 to 91% [39, 40]. Other programs in the US reported follow-up rates as low as 11% [35]. In one hearing screening program in the US conducted on four-year-old children, the follow-up rate was only 10% [41]. All hospital-based programs in India have reported 12 and 21% follow-up for 2nd screening and diagnostic assessment respectively [9, 12]. Though there are very few rural community-based studies, it is noteworthy that community clinic-based studies have shown high (91%) follow-up rates [31, 42], due to shorter travel.

The follow-up rate obtained in this program, irrespective of in-person or tele-ABR follow-up, is better than previous reports of follow-up rates in India, and is comparable to the high follow-up rates obtained in community clinic-based programs around the world. This suggests that in general, community-based programs have had greater success with follow-up. In this program, the improved follow-up compliance maybe attributed to the VHWs efforts in mobilizing and monitoring follow-up and therefore strongly supports a community-based model of hearing screening. Improved follow-up for tele-ABR in this program is comparable to the community clinic-based tele-diagnostic testing conducted in the Californian tele-audiology program [43].

The median number of days taken between 2nd screening and tele-ABR follow-ups were 30 days (10–189 days), and for in-person ABR follow-ups were 31 (30–36 days). The range for tele-ABR follow-up was wider. Tele-ABR was conducted once a month in the community, therefore, it was possible to achieve follow-ups as early as 10 days from the time of 2nd screening. Only one child was brought for tele-ABR after 6 months of 2nd screening, when the mother returned from her maternal home. Despite the minimal difference in the median time between the two follow-up methods, it was possible to achieve much earlier follow-ups for tele-ABR. In a mobile ear-screening service, the time between screening and tele-ENT evaluation consistently diminished over three years of the program. This suggests that with time, the tele- follow-up may show significant time gain [44].

According to Thompson et al., 2001, in the US, quality studies demonstrate that if 2041–2794 low-risk and 86–208 high-risk newborns were screened, then one case of moderate-to-profound permanent hearing loss was found. Though this program included children up to five years of age, four out of the 2815 screened were identified with moderate to profound hearing loss between three to five years of age and were recommended to partake in a hearing aid trial. Even though return rate for diagnostics were good, only two parents followed up for hearing aid trials and fitting. Poor follow-up for intervention can be attributed to a lack of awareness about the consequences of hearing loss, financial constraints in undertaking travel to the hospital to access rehabilitation services, and potential wage loss. Spivak et al., 2009, reported similar non-compliance rates for hearing aid fittings, particularly in infants with unilateral hearing loss.

## Conclusion

Non-availability of audiologists and limited infrastructure in rural areas has prevented the establishment of large scale hearing screening programs in India. In existing programs, considerable challenges with respect to follow-up for diagnostic testing was reported, due to travel requirements for accessing services and the potential in wage loss for doing so. In this community-based hearing screening program, tele-ABR improved follow-up rate when compared to in-person ABR.

In the absence of a systematic screening program for detection of permanent hearing loss in countries like India, at the time of program initiation it is worthwhile to include young children up to five years of age, as they benefit from early intervention. While this program was not designed to meet the JCIH benchmarks that are based on the hospital-based models of hearing screening established in western countries, the very low refer rate, and improved follow-up rates reflect the success of this community-based hearing screening program.

## Additional file

Additional file 1: Training evaluation form. This form describes the measures used to evaluate the performance of health workers. (DOCX 19 kb)

## Abbreviations

ABR: Auditory Brainstem Response
dB SNR: Decibel signal to noise ratio
dB SPL: Decibel sound pressure level
DPOAE: Distortion Product Oto-acoustic emissions
JCIH: Joint Committee on Infant Hearing
NGO: Non-governmental Organisation
RUWSEC: Rural Women’s Social Empowerment Centre
VHW: Village health worker

## Footnotes

1 Indian pre-school run for economically weaker sections of the society, either by government or NGOs .They are neighborhood schools and they have flexible school hours based on convenience of the community it serves.

2 Auditory Brainstem Response (ABR): a diagnostic test used to assess auditory evoked potential, obtained from the auditory nerve on acoustic stimulation.

3 Distortion Product Oto-acoustic emissions (DPOAE): a test used to assess outer hair cell function in the inner ear by recording emissions emanating from outer hair cell in response to acoustic stimulation. It is test routinely used for screening hearing.

4 Infants: Children under 1 year of age.

5 Young children: Children older than 1 year and under 5 years of age.

6 Balwadis are Indian pre-school run for economically weaker sections of the society, either by government or NGOs. They function during regular school hours and are centrally located to the community.
