# Supplementary material for: Implementation and evaluation of a rural community-based pediatric hearing screening program integrating in-person and tele-diagnostic auditory brainstem response (ABR)
Source: BMC Health Serv Res. 2019 Jan 3;19:1. doi: 10.1186/s12913-018-3827-x (PMC6318860; doi:10.1186/s12913-018-3827-x)
Supplement: Supplementary file 1 — Training evaluation form. This form describes the measures used to evaluate the performance of health workers. (DOCX 19 kb) [file 12913_2018_3827_MOESM1_ESM.docx]

**TRAINING EVALUATION FORM**

**Please read instruction for each item before you answer. Mark (🗸 ) on the appropriate choices wherever options are given. Write the answer wherever a blank space is provided.**

1. What is the earliest age at which hearing can be screened?

0-3months

3-6 months

6 months -1 year

1-3 years

3-6 years

>6 years

Don’t know

2. The age of identifying hearing loss is crucial.

Yes

No

Don’t know

Please mention, Why? ................................................................

3. Permanent hearing loss may occur due to damage to the ………………..

Outer ear

Middle ear

Inner ear

Don’t know

4. List some of the factors that may lead to hearing loss. (You can list as many as you know)

5. It is preferable to screen hearing in young babies by………..

Clapping hands and checking for baby’s response

OAE screening

ABR testing

Calling name and checking for baby’s response

Asking parent about child’s hearing

Don’t know

6. Have you heard about OAE screening?

Yes

No

7. OAE is used to screen function of……………

a. Outer ear

b. Middle ear

c. Inner ear

d. Don’t know

8. Identify the result of OAE screening.

a. Pass/ Fail

b. Normal/Abnormal

c. Pass/Refer

d. Don’t know

9. OAE screening should not be done when the baby …………&………….

a. Has fever

b. Has wax/ discharge in the ear canal

c. Is very young

c. Has severe cold

d. Don’t know

10. OAE screening:

a. Confirms presence/absence of hearing loss

b. Identifies those who need further test to rule out hearing loss

c. Can identify the degree of hearing loss

d. Don’t know

11. In very young babies, OAE screening should be conducted when baby is awake rather than asleep.

1. Yes
2. No
3. Don’t know

12. OAE screening should be conducted in a quiet place.

1. Yes
2. No
3. Don’t know

13. It is not preferable to conduct OAE screening when baby is being fed.

1. Yes
2. No
3. Don’t know

14. If a baby gets “Refer” in OAE screening, you should inform the parent/caregiver that:

a. Your baby has hearing loss

b. Your baby has failed the test

c. Your baby needs further testing

d. Don’t know

15. What will you do if the following message is displayed on the OAE equipment screen?

a. Probe is not completely sealed:

b. Test environment is too noisy:

**OSCE**

I. Spotters for parts of OAE- 10 points

II. Skill in OAE

1. Examines the ear to check if ear canal is clean- that it is free of infection/discharge/ any debris.
2. Chooses appropriate tip
3. Fixes tip to the probe and places in the ear canal
4. Presses “Test” button
5. Appropriately responds when the following occurs and completes the test

a.“Noisy”

b. Probe not completely sealed

1. Documents result of each frequency and overall result as soon as testing is completed for that ear.
2. Tests the other ear.
3. Cleans the probe tip after test completion
4. Counsels the mother appropriately about the test result of “pass” and “refer”.

III. Skill in assisting in ABR

1. Asks the mother/ caregiver to seat the child on the lap or bed
2. Uses sufficient cleaning gel on cotton
3. Rubs the 4 sites: upper forehead, lower forehead, and the two mastoids
4. Removes plastic covering from the electrode disc
5. Places the disc and ensures it is well adhering to the skin (no hair caught between skin and electrode
6. Places insert earphones with ear tip in the babies ear and ensures deep insertion
7. Achieves impedance <5kohms
